# Invitation strategies and participation in a community-based lung cancer screening programme located in areas of high socioeconomic deprivation

**DOI:** 10.1136/thorax-2023-220001

**Published:** 2023-08-16

**Authors:** Patrick Goodley, Haval Balata, Alberto Alonso, Christopher Brockelsby, Matthew Conroy, Nicola Cooper-Moss, Christopher Craig, Matthew Evison, Kath Hewitt, Coral Higgins, William Johnson, Judith Lyons, Zoe Merchant, Ailsa Rowlands, Anna Sharman, Nicola Sinnott, Matthew Sperrin, Richard Booton, Philip A J Crosbie

**Affiliations:** 1 Division of Immunology, Immunity to Infection and Respiratory Medicine, The University of Manchester, Manchester, UK; 2 Manchester Thoracic Oncology Centre (MTOC), Manchester University NHS Foundation Trust, Manchester, UK; 3 Manchester Integrated Care Partnership (NHS Greater Manchester), Manchester, UK; 4 Centre for Primary Care, The University of Manchester, Manchester, UK; 5 Faculty of Biology Medicine and Health, The University of Manchester, Manchester, UK; 6 Division of Informatics Imaging and Data Sciences, The University of Manchester, Manchester, UK

**Keywords:** Lung Cancer, Clinical Epidemiology

## Abstract

**Introduction:**

Although lung cancer screening is being implemented in the UK, there is uncertainty about the optimal invitation strategy. Here, we report participation in a community screening programme following a population-based invitation approach, examine factors associated with participation, and compare outcomes with hypothetical targeted invitations.

**Methods:**

Letters were sent to all individuals (age 55–80) registered with a general practice (n=35 practices) in North and East Manchester, inviting ever-smokers to attend a Lung Health Check (LHC). Attendees at higher risk (PLCO_m2012NoRace_ score≥1.5%) were offered two rounds of annual low-dose CT screening. Primary care recorded smoking codes (live and historical) were used to model hypothetical targeted invitation approaches for comparison.

**Results:**

Letters were sent to 35 899 individuals, 71% from the most socioeconomically deprived quintile. Estimated response rate in ever-smokers was 49%; a lower response rate was associated with younger age, male sex, and primary care recorded current smoking status (_adj_OR 0.55 (95% CI 0.52 to 0.58), p<0.001). 83% of eligible respondents attended an LHC (n=8887/10 708). 51% were eligible for screening (n=4540/8887) of whom 98% had a baseline scan (n=4468/4540). Screening adherence was 83% (n=3488/4199) and lung cancer detection 3.2% (n=144) over 2 rounds. Modelled targeted approaches required 32%–48% fewer invitations, identified 94.6%–99.3% individuals eligible for screening, and included 97.1%–98.6% of screen-detected lung cancers.

**Discussion:**

Using a population-based invitation strategy, in an area of high socioeconomic deprivation, is effective and may increase screening accessibility. Due to limitations in primary care records, targeted approaches should incorporate historical smoking codes and individuals with absent smoking records.

**WHAT IS ALREADY KNOWN ON THIS TOPIC**

- Lung cancer screening reduces disease-specific mortality; however, the optimal approach to invitation is unclear. Primary care records can be used to identify and target people who have smoked, but this risks missing individuals whose smoking status is not known or is inaccurately recorded.

**WHAT THIS STUDY ADDS**

- We demonstrate that a population approach to screening invitation, in an area of high smoking prevalence and socioeconomic deprivation, is effective and overcomes limitations in primary care smoking records. However, our modelling suggests ever-smokers likely not identified by targeted approaches are generally at lower risk of lung cancer.

**HOW THIS STUDY MIGHT AFFECT RESEARCH, PRACTICE OR POLICY**

- This study highlights inaccuracies in primary care smoking records and strategies to more robustly identify people who have ever smoked. This may either be through a population invitation approach or an optimised targeted approach, which includes live and historic smoking codes and those with absent smoking records.

## Introduction

Targeted low-dose CT (LDCT) screening reduces lung cancer mortality.^1 2^ Screening was first recommended in the USA in 2013.^3^ However, despite widespread screening availability and public health insurance coverage, uptake has been reported to be as low as 2%.^4 5^ In England, following the success of local initiatives, lung cancer screening implementation is expanding through the Targeted Lung Health Check (TLHC) programme.^6–9^ The UK National Screening Committee recently recommended national adoption of lung cancer screening, using the TLHC model, following a favourable cost-effectiveness evaluation.^10^

An important question for screening implementation is how best to identify and invite the target group. The national TLHC protocol recommends inviting ever-smokers (current or former) aged 55–74, but no specific strategy is stipulated for determining smoking status from healthcare records or otherwise.^11^ Two main approaches are possible. A ‘*targeted*’ invitation approach uses primary care records to identify and invite only individuals with a record of having smoked. Variations of this approach were used in the Lung Screen Uptake Trial (LSUT), SUMMIT Study, and Yorkshire Lung Screening Trial (YLST).^8 12 13^ It relies on smoking records being sufficiently comprehensive and accurate. There is limited understanding of smoking code reliability in the wider population, including those recorded as never-smokers. The UK Clinical Practice Research Datalink and the Welsh Secure Anonymised Information Linkage databases report a 2%–6% missing smoking record rate.^14 15^ However, there is uncertainty regarding the accuracy of existing records and whether practices not contributing to research databases have higher levels of missing or inaccurate data. SUMMIT’s targeted approach found discordance between primary care code and self-reported smoking status in a quarter of invitees.^16^ Alternatively, a ‘*population*’ invitation approach sends letters to everyone within the eligible age range, offering an LHC to those who have ever smoked. Such a strategy was used in the UK Lung Screening trial (UKLS).^17^ This reduces the risk of not inviting eligible individuals due to missing or inaccurate primary care records, but is associated with a higher cost due to the larger number of letters posted. Additionally, it is not known whether receiving an invitation brings harm, such as undue anxiety to never-smokers given that they are not eligible for screening by contemporary criteria.

LHCs, offering community-based lung cancer screening using a mobile LDCT scanner, were first piloted in Manchester in 2016.^7^ In 2019, the service expanded to cover the whole of North and East Manchester (NEM), an area of high socioeconomic deprivation. Letters were posted to everyone in the eligible age range inviting ever-smokers to book a free LHC. We hypothesised this would increase service accessibility by including individuals with inaccurate or missing primary care smoking records. In this study, we report screening outcomes following our population-based invitation strategy including participation, adherence, and lung cancer detection. We also model screening participation and cancer detection according to hypothetical targeted invitation strategies.

## Methods

### Study design

This prospective cohort study analysed participation, second round screening adherence, and lung cancer detection in a community-based LHC service following a population-based invitation approach. All individuals who received at least one LHC letter were included in the analysis. Individuals were excluded if the invitation was returned to sender. Outcomes were stratified according to three hypothetical targeted invitation strategies based on the primary care recorded smoking status.

### Lung Health Check

The North and East Manchester LHC (NEM-LHC) programme was similar in design to our previous pilot.^6 7^ In brief, individuals aged 55–80 years who had ever smoked and were registered with a general practice (GP) (n=35) in North and East Manchester were eligible for an LHC. The LHC was nurse-led and located in convenient community settings. It comprised a symptom check, spirometry, stop smoking support, cardiovascular and lung cancer risk assessment. The PLCO_m2012NoRace_ risk prediction tool was used for risk assessment. This multivariable model predicts the risk of being diagnosed with lung cancer within 6 years, without screening.^18^ Those at higher risk, using a threshold of ≥1.5%, were eligible for annual LDCT screening over two rounds with immediate access to a mobile CT scanner. The programme commenced in April 2019. Due to the COVID-19 pandemic, the start of the second screening round was delayed by 4 months, from April to July 2020.

### Primary care recorded smoking status

Data extracted from primary care for invitees included age, sex, and smoking status codes. Index of Multiple Deprivation (IMD) was determined using postcodes.^19^ Smoking status codes (current or former) were clustered into ever-smoking categories according to NHS Digital’s Quality Outcomes Framework (see online supplemental appendix table S1).^20^ The smoking code present on 31 January 2019 was denoted as the ‘live’ code. All previous smoking status codes, with dates, were extracted and used to define ‘historical’ smoking status. Each person therefore had a ‘live’ and ‘historical’ ever-smoking status. If the medical record was accessible but no smoking status was recorded at any time, this was categorised as ‘absent’. To model targeted invitation strategies, we categorised invitees into one of four smoking status groups, defined as follows: *group 1*: individuals with an ever-smoker live code (live ever, historical any); *group 2*: individuals with a live never-smoking code but at least one historical ever-smoking code (live never, historical ever); *group 3*: individuals coded as never-smokers in the live and all historical smoking codes (live never, historical never) and *group 4*: individuals with no live or historical smoking codes recorded (live absent, historical absent). Self-reported smoking status was recorded for all invitees who responded. This was compared with the primary care record.

10.1136/thorax-2023-220001.supp1 Supplementary data

### Invitation strategies

GP-endorsed letters were sent to all individuals, registered with a participating GP (n=35), in the eligible age range. These invited ever-smokers (current or former) to contact a telephone number to book an LHC appointment. Up to two reminder letters were sent to non-respondents. We termed this a ‘*population*’ invitation approach. We compared this to three hypothetical ‘*targeted*’ invitation strategies, which we defined as follows: *strategy 1*: invite only those with a live ever-smoking code (group 1); *strategy 2*: invite those with any ever-smoking code, even if the historic code had been succeeded by a never-smoker code (groups 1 and 2) and *strategy 3*: invite those with any ever-smoking code and individuals with no smoking code recorded (groups 1, 2 and 4). For each of these targeted approaches, the number of invitations, responses, LHC attendees, baseline LDCT scans performed and lung cancer diagnoses (after two screening rounds) were estimated by assuming that each invitee would have responded to their invitation in the same way as they did with the population approach.

### Screening participation and adherence

Screening participation and outcomes were collected in a clinical service database for all participants who had at least one LDCT scan. Lung cancer cases were those detected through screening and confirmed by the lung cancer multidisciplinary team. The response rate was defined as the number of eligible individuals (self-reported ever-smokers) contacting the LHC telephone line divided by the number of ever-smokers in primary care records. This denominator was used to enable comparison of response rates between this programme and others, which mostly send invitation letters to individuals identified as having smoked from primary care records. LHC uptake was defined as the proportion of respondents who self-reported being ever-smokers and had an LHC. Baseline round attendees were eligible for the second screening round 1 year later unless they were diagnosed with lung cancer, developed comorbidity preventing them from participating, died, or moved out of the area. Screening adherence was defined as the proportion of eligible screenees returning for the second screening round. Participants who returned for their next screen were considered adherent even if their scan was delayed. This was due to the disruption caused by the COVID-19 pandemic.

### Statistical analysis

Welch two sample t-test was used for continuous variables with symmetric distribution, Wilcoxon rank sum test for continuous non-symmetric variables and Pearson’s χ^2^ test for categorical variables. Means are reported with SDs (±SD) and medians with IQRs (Q1–Q3). Univariable and multivariable logistic regression was also applied to estimate the association between available variables and participation. Multivariable models were adjusted for age and sex. ORs are presented with 95% CIs. Statistical analysis was performed using R V.4.1 with the gtsummary package.^21^

## Results

### Population invitation strategy

**Figure 1:**
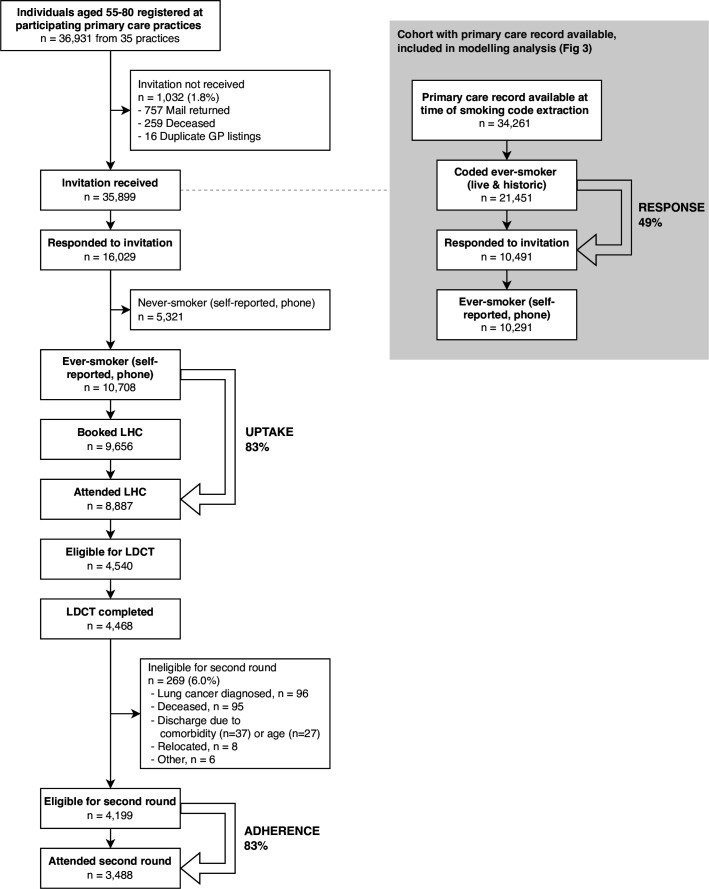
Flow diagram of invitees through the North and East Manchester Lung Health Check programme, showing participation at each stage. The subset of invitees for whom full smoking status codes were available is offset on the right. GP, general practice; LDCT, low-dose CT; LHC, Lung Health Check.

A total of 35 899 individuals in North and East Manchester received an LHC invitation letter (figure 1). Median invitee age was 64 years (IQR 59–70), 49% were women, and 71% (n=25 559) from the most socioeconomically deprived quintile (table 1). Overall, 16 029 people responded by contacting the LHC telephone line. Two-thirds self-reported being an ever-smoker (n=10 708) and one-third a never-smoker (n=5321). Never-smokers were ineligible for LHCs. The proportion of eligible respondents who booked an LHC appointment was 90% (n=9656/10 708); of these, 92% attended (n=8887/9656). The overall LHC uptake rate was 83% (n=8887/10 708). Just over half of LHC attendees were eligible for screening (51%, n=4540/8887), 98% of whom had a baseline LDCT scan (n=4468/4540). Out of 4199 participants eligible for the second round, 83% (n=3488) attended. The median interval between baseline and second round screens was 12.9 months (IQR 12.0–14.3), with 98.4% (n=3421) performed within 6 months of a true 12 month interval. None of 67 participants screened later than this were diagnosed with lung cancer. Over two screening rounds, 3.2% (n=144) were diagnosed with lung cancer.

**Table 1:** Characteristics and participation among the population sent invitations for the North and East Manchester Lung Health Check programme

Variable	N=35 899
Age (years), median (IQR)	64 (59–70)
Age strata, n (%)	
55-60	10 738 (30)
60–64	8591 (24)
65–69	6594 (18)
70–74	5556 (16)
≥75	4420 (12)
Female, n (%)	17 578 (49)
Index of Multiple Deprivation decile, n (%)	
1	19 643 (55)
2	5916 (17)
3	3828 (11)
4	2389 (6.7)
5	1778 (5)
6	971 (2.7)
7	608 (1.7)
8	251 (0.7)
9	501 (1.4)
10	14 (0)

### Primary care recorded smoking status

We analysed primary care smoking status in invitees with available primary care records (95% of all invitees, n=34 261/35 899) and compared this to self-reported smoking status. Based on the ‘live’ (or most recent) smoking code more than half of invitees were coded as ever-smokers (52%, n=17 895), 43% as never-smokers (n=14 558) and 5.3% had absent smoking codes (n=1808). A quarter of those with a live never-smoker code had at least one historical code indicating previous smoking (24%, n=3556), suggesting their live code was incorrect. Overall, 11% of respondents to the LHC invitation who had at least one ever-smoking code in their primary care record reported being a never-smoker (n=1123/10 491). This discordance varied from 2.6% (n=227/8686) in those with a live ever-smoking code to 49.6% (n=896/1805) in those with a historical ever-smoking code only (p<0.0001). In respondents coded as never-smokers, 16% self-reported being an ever-smoker (n=738/4616). Almost half of respondents with absent smoking codes self-reported being ever-smokers (48%, n=209/438). The presence of any discordance between primary care recorded and self-reported ever-smoking status ranged from 5.7% to 22% (median 11%) in participating GPs (n=35), a fourfold variation (further detail in online supplemental S2 and table S2a,2b).

### Smoking status groups

**Figure 2:**
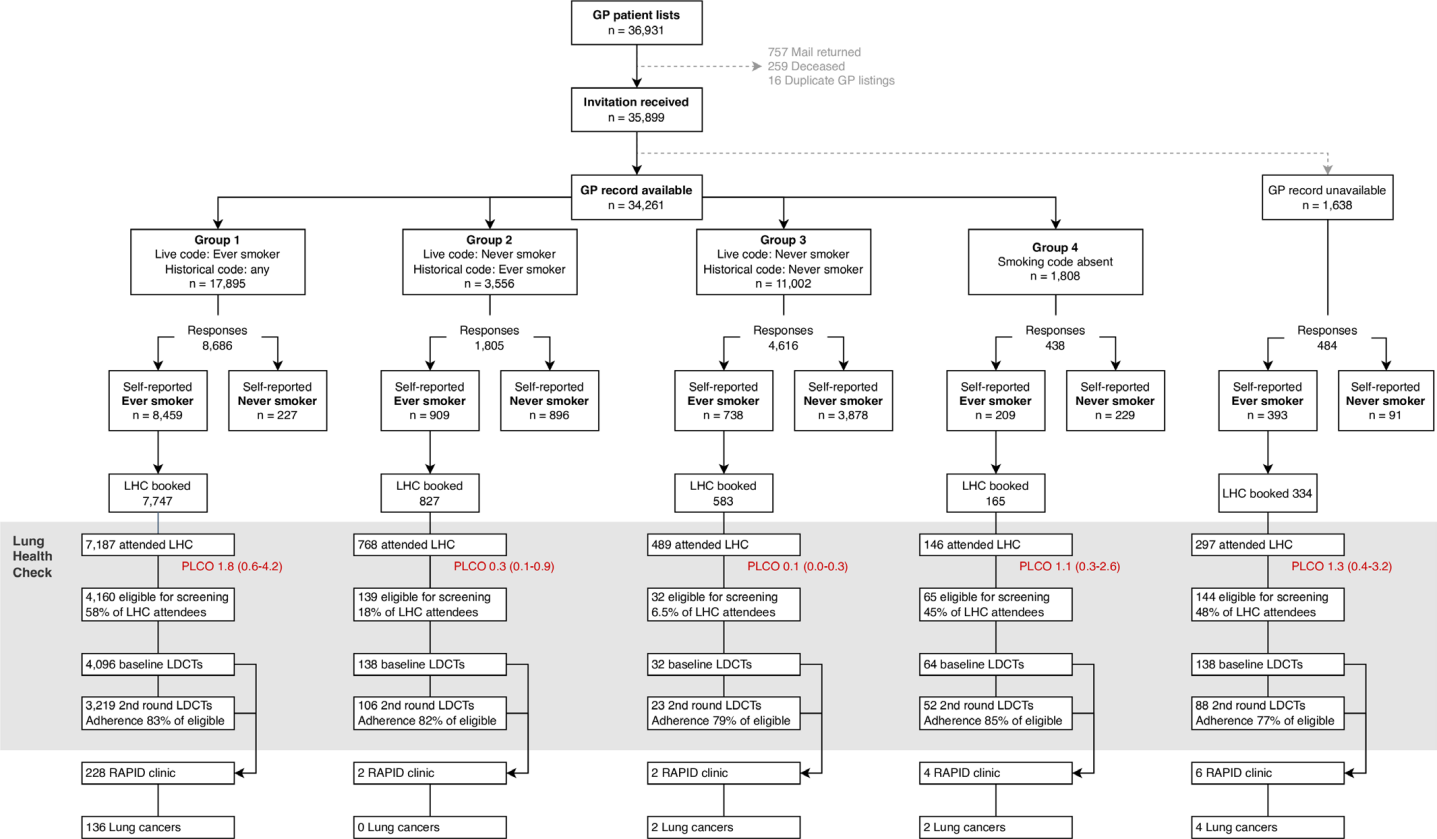
Primary care (GP) smoking status records among invitees to NEM-LHC programme and screen-detected lung cancer cases during the first two rounds. PLCO refers to PLCO_m2012norace_, with median (IQR) given. RAPID clinic is the fast-track lung cancer service at the Manchester Thoracic Oncology Centre. GP, general practice; LDCT, low-dose CT; LHC, Lung Health Check; RAPID, Rapid Access to Pulmonary Investigation and Diagnosis.

LHC invitees were categorised into one of four groups based on live and historical primary care recorded ever-smoking codes. The characteristics of each group are detailed in table 2 and figure 2. In LHC attendees, average lung cancer risk scores were highest in group 1, individuals with a live ever-smoker code (n=7187, median PLCO_m2012NoRace_ 1.8%, IQR 0.6–4.2) and lowest in group 3, those coded as never-smokers (n=489, median PLCO_m2012NoRace_ 0.1%, IQR 0–0.3). The proportion of LHC attendees eligible for screening was 58% in individuals with a live ever-smoking code (group 1; n=4160/7187), 45% in those with absent smoking codes (group 4; n=65/146), 18% in ever-smokers with ‘historical’ ever-smoking codes only (group 2, n=139/768) and 6.5% in never-smokers (group 3; n=32/489). The likelihood of an invitation leading to an individual attending an LHC and being eligible for screening was significantly greater for ever-smokers with a ‘live’ code compared with an ever-smoker with a ‘historical’ code only (OR 7.5 (95% CI 6.3 to 8.9), p<0.0001). The vast majority of lung cancers were diagnosed in individuals with ‘live’ ever-smoking codes (group 1) (97.1%, n=136/140).

**Table 2:** Participation and baseline characteristics of invitees with available primary care records (n=34 261), stratified by smoking status group

Primary care smoking status	Group 1	Group 2	Group 3	Group 4
Live code	Ever	Never	Never	Absent
Historical code	Any	Ever	Never	Absent
Invitees, n (%)	17 895 (52.2)	3556 (10.4)	11 002 (32.1)	1808 (5.3)
Outcomes, n (%)				
Responded	8686 (48.5)	1805 (50.8)	4616 (42)	438 (24.2)
Self-reported ever-smoker	8459 (47.3)	909 (25.6)	738 (6.7)	209 (11.6)
Booked LHC	7747 (43.3)	827 (23.3)	583 (5.3)	165 (9.1)
Attended LHC	7187 (40.2)	768 (21.6)	489 (4.4)	146 (8.1)
Eligible for LDCT	4160 (23.2)	139 (3.9)	32 (0.3)	65 (3.6)
LDCT completed	4096 (22.9)	138 (3.9)	32 (0.3)	64 (3.5)
Screening result positive	228 (1.3)	2 (0.1)	2 (0)	4 (0.2)
Screen-detected lung cancer	136 (0.8)	0 (0)	2 (0)	2 (0.1)
Invitee characteristics				
Age (years), median (IQR)	64 (59–71)	65 (59–72)	63 (58–69)	63 (58–69)
Female, n (%)	8195 (45.8)	1832 (51.5)	6051 (55)	806 (44.6)
Self-reported smoking status, n (% of ever-smoker respondents)				
Former	5773 (68.2)	813 (96.3)	573 (94.7)	133 (77.3)
Current	2686 (31.8)	31 (3.7)	32 (5.3)	39 (22.7)
LHC attendee characteristics (n=8590)				
Cigarettes per day, median (IQR)	20 (10–21)	10 (5–20)	6 (3–15)	20 (10–25)
Smoking pack years, median (IQR)	32 (16–48)	10 (2–23)	3 (1–10)	24 (10–44)
Quit duration (years), median (IQR)	8 (0–23)	30 (18–40)	34 (20–42)	16 (1–30)
PLCO_m2012norace_ (%), median (IQR)	1.8 (0.6–4.2)	0.3 (0.1–0.9)	0.1 (0.0–0.3)	1.1 (0.3–2.6)

LDCT, low-dose CT; LHC, Lung Health Check.

### Targeted invitation strategies

**Figure 3:**
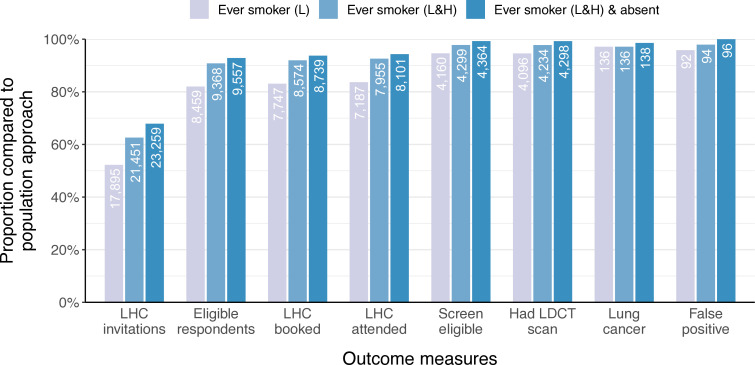
Comparison of the impact of three invitation strategies along the screening pathway, and key screening outcomes, compared with population invitation strategy used in North & East Manchester. ‘L’ refers to live smoking status codes while ‘H’ refers to historical smoking status codes. LDCT, low-dose CT; LHC, Lung Health Check.

We modelled three targeted invitation strategies and assessed outcomes relative to the population approach (see figure 3 and online supplemental table S3), as detailed below.

- *Strategy 1*: The most stringent method of targeted invitation was based on live codes denoting ever-smoker status only (group 1; n=17 895). This was estimated to require 48% fewer invitations, yielding 18% fewer eligible respondents and 16% fewer LHC attendees. We estimate that 5.4% fewer people would have been eligible for screening and 2.9% (n=4/140) fewer individuals would have been diagnosed with screen-detected lung cancer.
- *Strategy 2*: Broadening invitations to include those with any ever-smoker code, live or historical (groups 1 and 2; n=21 451), would require 37% fewer invitations, result in 9.2% fewer eligible respondents and 7.4% fewer LHCs. We estimate a 2.2% reduction in individuals eligible for screening and 2.9% (n=4/140) reduction in screen-detected lung cancer.
- *Strategy 3*: The most permissive approach, which extended strategy 2 by also sending letters to those with absent smoking codes (groups 1,2 and 4; n=23 259), was estimated to require 32% fewer invitations, leading to 7.2% fewer ever-smoker respondents and 5.7% fewer LHCs. The estimated reduction in those eligible for screening was 0.7%, with 1.4% (n=2/140) fewer individuals diagnosed with screen-detected lung cancer.

Overall LHC uptake was similar irrespective of invitation strategy (population 83% vs targeted 85%).

### Screening participation and adherence

Among 34 261 invitees with primary care records available, there were 21 451 coded ever-smokers and their response rate was 49% (n=10 491/21 451). Factors associated with response to invitation, LHC uptake, and screening adherence are shown in table 3. The strength of association between invitee characteristics and participation at each step is shown in table 4. There was a significant difference in response rate by primary care recorded smoking status, with 56% (n=6938/12 433) of individuals coded as former smokers responding compared with only 39% (n=3553/9018) of those coded as current smokers (_adj_OR 0.55 (95% CI 0.52 to 0.58), p<0.001). Other variables associated with reduced response rate included younger age (41%, age 55–59 vs 56%, age 75–80), socioeconomic deprivation, and male sex.

**Table 3:** Characteristics of people invited to the North and East Manchester Lung Health Check programme, stratified by participation at each stage

Stage of participation Cohort description	Response to invitation Invitees with available general practice records with an ever-smoking status (live or historic, N=21 451), stratified by telephone response to invitation letter			LHC uptake Respondents who self-reported as ever-smokers (N=10 708), stratified by attendance at baseline Lung Health Check			Adherence at second screening round Participants eligible to continue screening (N=4199), stratified by attendance at the next annual round		
	Responded	Did not respond	P value*	Attended LHC	Did not attend LHC	P value*	Attended second round	Did not attend second round	P value^*^
n (%)	10 491 (49%)	10 960 (51%)		8887 (83%)	1821 (17%)		3488 (83%)	711 (17%)	
Age strata, n (%)			<0.001			<0.001			<0.001
55–59	2477 (24%)	3513 (32%)		2170 (24%)	437 (24%)		601 (17%)	168 (24%)	
60–64	2259 (22%)	2630 (24%)		2025 (23%)	307 (17%)		794 (23%)	158 (22%)	
65–69	2172 (21%)	1921 (18%)		1874 (21%)	304 (17%)		849 (24%)	143 (20%)	
70–74	2014 (19%)	1655 (15%)		1689 (19%)	318 (17%)		771 (22%)	134 (19%)	
75–80	1569 (15%)	1241 (11%)		1129 (13%)	455 (25%)		473 (14%)	108 (15%)	
Sex, n (%)			<0.001			0.001			0.47
Female	5040 (48%)	4987 (45%)		4131 (46%)	921 (51%)		1651 (47%)	347 (49%)	
Male	5451 (52%)	5973 (55%)		4756 (54%)	900 (49%)		1837 (53%)	364 (51%)	
Index of Multiple Deprivation quintile, n (%)			<0.001			<0.001			0.001
1 (most deprived)	7393 (70%)	8281 (76%)		6180 (70%)	1372 (75%)		2580 (74%)	575 (81%)	
2	1916 (18%)	1694 (15%)		1643 (18%)	288 (16%)		599 (17%)	86 (12%)	
3	813 (8%)	679 (6%)		729 (8%)	105 (6%)		220 (6%)	31 (4%)	
4	232 (2%)	198 (2%)		211 (2%)	33 (2%)		63 (2%)	11 (2%)	
5 (least deprived)	137 (1%)	108 (1%)		124 (1%)	23 (1%)		26 (1%)	8 (1%)	
Smoking status, n (%)†			<0.001			<0.001			<0.001
Current	3553 (34%)	5465 (50%)		2366 (27%)	521 (34%)		1482 (42%)	369 (52%)	
Former	6738 (66%)	5495 (50%)		6521 (73%)	1019 (66%)		2006 (58%)	342 (48%)	
Education level, n (%)									0.87
No school qualifications	–	–		–	–		2513 (73%)	514 (74%)	
O-level or equivalent	–	–		–	–		353 (10%)	68 (10%)	
A-level or equivalent	–	–		–	–		51 (1%)	10 (1%)	
Some further education	–	–		–	–		380 (11%)	75 (11%)	
Undergraduate	–	–		–	–		97 (3%)	16 (2%)	
Postgraduate	–	–		–	–		23 (1%)	5 (1%)	
Prefer not to say or missing							71 (2%)	23 (3%)	
PLCO_m2012NoRace_ (%), median (IQR)	–	–		–	–		3.4 (2.2–6.2)	3.7 (2.2–6.4)	0.24
Chronic obstructive pulmonary disease, n (%)	–	–		–	–		432 (12%)	102 (14%)	0.15
FEV_1_ % predicted, mean (±SD)	–	–		–	–		61 (±16)	59 (±17)	0.010

*Welch two sample t-test; Pearson’s χ^2^ test; Wilcoxon rank sum test.

†Smoking status for response is based on the primary care codes, whereas smoking status for uptake and adherence is self-reported. N=281 invitees who did not attend LHC did not have current/former status detail, so are missing.

LHC, Lung Health Check.

**Table 4:** Association between invitee characteristics and participation through the North and East Manchester Lung Health Check programme

	Response to invitation Odds of response to invitation among general practice-recorded ever-smokers		LHC uptake Odds of ever-smoking respondents attending LHC		Adherence to screening Odds of eligible screenees attending second round	
	Univariable OR (95% CI)	P value	Univariable OR (95% CI)	P value	Univariable OR (95% CI)	P value
Age strata						
55–59	—		—		—	
60–64	1.22 (1.13 to 1.31)	<0.001	1.20 (1.01 to 1.44)	0.037	1.40 (1.10 to 1.79)	0.006
65–69	1.60 (1.48 to 1.74)	<0.001	1.11 (0.93 to 1.32)	0.24	1.66 (1.30 to 2.12)	<0.001
70–74	1.73 (1.59 to 1.88)	<0.001	0.96 (0.81 to 1.15)	0.68	1.61 (1.25 to 2.07)	<0.001
75–80	1.79 (1.64 to 1.96)	<0.001	0.52 (0.44 to 0.61)	<0.001	1.22 (0.94 to 1.61)	0.14
Sex						
Male	—		—		—	
Female	1.11 (1.05 to 1.17)	<0.001	0.85 (0.77 to 0.94)	0.001	0.94 (0.80 to 1.11)	0.47
Index of Multiple Deprivation quintile						
1 (most deprived)	—		—		—	
2	1.27 (1.18 to 1.36)	<0.001	1.27 (1.10 to 1.46)	<0.001	1.55 (1.22 to 1.99)	<0.001
3	1.34 (1.21 to 1.49)	<0.001	1.54 (1.25 to 1.92)	<0.001	1.58 (1.09 to 2.37)	0.02
4	1.31 (1.08 to 1.59)	0.006	1.42 (0.99 to 2.09)	0.065	1.28 (0.70 to 2.57)	0.46
5 (least deprived)	1.42 (1.10 to 1.83)	0.007	1.20 (0.78 to 1.92)	0.43	0.72 (0.34 to 1.72)	0.43
Smoking status*						
Former	—		—		—	
Current	0.51 (0.49 to 0.54)	<0.001	0.71 (0.63 to 0.80)	<0.001	0.68 (0.58 to 0.80)	<0.001
**IMD quintile**	**Age–sex-adjusted OR**	**P value**	**Age–sex-adjusted OR**	**P value**	**Age–sex-adjusted OR**	**P value**
1 (most deprived)	—		—		—	
2	1.25 (1.16 to 1.34)	<0.001	1.28 (1.12 to 1.47)	<0.001	1.52 (1.20 to 1.96)	<0.001
3	1.31 (1.18 to 1.46)	<0.001	1.57 (1.27 to 1.95)	<0.001	1.53 (1.06 to 2.30)	0.031
4	1.27 (1.04 to 1.54)	0.017	1.49 (1.04 to 2.21)	0.036	1.32 (0.72 to 2.67)	0.4
5 (least deprived)	1.32 (1.02 to 1.70)	0.036	1.25 (0.81 to 2.02)	0.33	0.68 (0.32 to 1.63)	0.35
Smoking status*						
Former	—		—		—	
Current	0.55 (0.52 to 0.58)	<0.001	0.66 (0.59 to 0.74)	<0.001	0.71 (0.60 to 0.85)	<0.001

*Smoking status for response is based on the primary care codes, whereas smoking status for uptake and adherence is self-reported

adjOR, adjusted for age and sex; LHC, Lung Health Check.

Among eligible respondents who were offered an LHC appointment, self-reported current smokers were less likely to attend than former smokers (_adj_OR 0.66 (95% CI 0.59 to 0.74), p<0.001). Self-reported current smokers were also at much higher risk of lung cancer than former smokers, with median PLCO_m2012NoRace_ scores of 3.5% (1.8%–7.2%) and 0.9% (0.3%–2.5%) (p<0.001), respectively, despite being younger (median age 62 vs 67, p<0.001). There was also a significantly higher rate of screen detected lung cancer among current smokers (2.7%, n=65/2366 vs 1.2%, n=79/6519; p<0.0001). Lower LHC uptake was also associated with female sex (OR 0.85 (95% CI 0.77 to 0.94), p=0.001), socioeconomic deprivation, and younger age.

Screening adherence in those eligible for a second round LDCT scan was significantly lower in self-reported current smokers (_adj_OR 0.71 (95% CI 0.60 to 0.85), p<0.001). Adherence was also lower in younger participants and those with higher PLCO_m2012NoRace_ risk scores (_adj_OR 0.98 per percentage point increase, p=0.04). Education level or having a self-reported pre-existing diagnosis of COPD was not significantly associated with adherence (p=0.87 and p=0.15, respectively).

### Discussion

In this study, we report participation in a community lung cancer screening programme following a population-based invitation approach (n=35 899). An estimated 49% of ever-smokers responded to the LHC invitation. Out of 10 708 eligible respondents, 83% (n=8887) had an LHC, 42% (n=4540) were eligible for screening, and 42% had a baseline LDCT scan (n=4468). Second round screening adherence was 83%. After two rounds of screening, 3.2% (n=144) were diagnosed with lung cancer. We observed significantly lower screening participation in primary care recorded current smokers, younger individuals (age 55–59) and those in the most socioeconomically deprived quintile for all aspects of the programme (response rate, LHC uptake and screening adherence). These observations are consistent with those reported in other studies.^8 22–24^ Men had significantly lower response rates, but, interestingly, those who did respond were more likely to attend an LHC than women who responded.

Modelling suggested targeted strategies would have required 32%–48% fewer invitation letters, reduced the number of LHCs performed by 5.7%–16%, resulted in 0.7%–5.4% fewer people screened, and missed 1.4%–2.9% of lung cancers over two screening rounds. The optimal targeted approach included ever-smokers based on live and historical smoking codes as well as those with absent smoking codes. We identified inaccuracies in primary care recorded smoking status. For instance, one quarter of those with a live never-smoker code had historical statuses consistent with previous smoking, with significant variation seen between GP practices. There was also a significant discrepancy between self-reported smoking status and that recorded in primary care. The proportion of invitees coded as never-smokers who self-reported ever smoking ranged from 4.9% in strategy 2 (live and historical codes) to 11% in strategy 1 (live code only). These individuals would not have been invited to an LHC using a targeted approach. Our data address a research gap identified by an analysis in the SUMMIT trial, which sent letters to people with a ‘current’ smoking code entered in the previous 20 years.^16^ With this approach, coded never-smokers and those with no smoking code at all were not sent invitation letters, and therefore did not have the opportunity to self-report as ever-smokers. In NEM-LHC, 37% of invitees (n=12 828/34 261) were in these categories, with 7.4% of them (n=947) self-reporting being ever-smokers (table 2 and figure 2). Respondents with absent smoking codes had notably higher tobacco exposure, and higher lung cancer risk scores, than those with never-smoking codes or historical ever-smoking codes. This may imply that the absence of any smoking record does not reflect low smoking exposure or lung cancer risk, and therefore this group ought to be included in any targeted invitation strategy. Furthermore, the absence of smoking codes may reflect lower general engagement with health services and therefore fewer opportunities for smoking status to be captured.

We report an estimated response rate of 49% in ever-smokers. This is similar to LSUT (53%) and YLST (51%), which used targeted invitations. In UKLS, which used a population approach, 31% of invitees responded positively to invitation. All three studies also reported reduced participation associated with socioeconomic deprivation and current smoking status.^8 22 24^ The median socioeconomic status of UKLS and YLST invitees was in the third (‘average’) quintile (based on IMD), compared with the lowest quintile in the NEM-LHC cohort. Our study therefore provides novel insights into screening participation in a more socioeconomically disadvantaged population.

The consistent finding that current smokers are less likely than former smokers to engage in LHCs or screening is concerning, particularly given their higher lung cancer risk. This phenomenon has been explored by Quaife *et al*, who prospectively demonstrated that current smokers perceive excessive fatalism about their ability to reduce their lung cancer risk and have lower confidence in the benefit of available treatments if they were diagnosed.^25^ These perceptions lower current smokers’ willingness to participate in screening. Further work to address this challenge would likely translate into greater population benefit from screening.

The previous Manchester LHC pilot saw 90% second-round adherence.^6^ This was higher than the 83% observed in NEM-LHC, where the second round coincided with the COVID-19 pandemic in 2020. This may have discouraged adherence for a proportion of screenees, although subjective reasons for non-adherence were not captured systematically. A meta-analysis of cohort studies in the USA reported pooled adherence of 57%, with younger participants and current smokers also at particular risk of non-adherence.^26^ The higher overall uptake and adherence rates seen in the Manchester may in part be attributable to systematic invitation and the convenience of community-based screening units.^27^

The results generated from this study are of direct relevance for screening implementation, and importantly engaged individuals from areas of high socioeconomic deprivation, who are often under-represented in trial populations. Self-reported smoking status was taken as ground truth for the purposes of eligibility for LHCs, but this is fallible. Previous or light tobacco exposures may be misreported. It is possible that some individuals could alter their smoking history to render themselves either eligible or ineligible for screening, or to avoid perceived stigma of being labelled a ‘smoker’.^28^ Response rates were estimated based on the primary care smoking codes, which may be imprecise. Smoking codes are entered to reflect smoking history at the time of entry, but smoking habits change over time and this may underlie some of the discordance with self-reported status at the point of LHC invitation. Our modelled comparison of invitation strategies assumed that invitees would have behaved identically if targeted approaches had been used. It is possible that LHC participation would have differed if screening behaviour were influenced by peers or relations who received, or did not receive, invitations. As only two rounds of screening were completed, we could not explore participation in subsequent rounds or lung cancer detection over the longer term.

Prospective studies comparing varied invitation strategies could provide further important refinements to programme delivery. A pragmatic adaptation of population-based invitation would be to avoid reminder letters for non-respondents who have a never-smoking primary care record. Further research is needed to explore ways of improving current smoker engagement in screening, and whether a response to telephone triage is the same irrespective of smoking status. Another potential modification could see current smokers invited to an LHC regardless of screening eligibility, where they might benefit from smoking cessation interventions, while using telephone triage for former smokers. Additionally, further understanding of the extent, and reasons for, variation in the completeness and accuracy of primary care smoking records could point to opportunities for improvement. Interventions, such as text message-based updating of smoking status records, have been explored with promising findings. A Welsh study found that sending automated messages to primary care patients with no smoking status recorded resulted in 57% of recipients responding to declare their smoking status.^29^ Existing online tools, such as the National Health Service (NHS) app,^30^ could be leveraged for this purpose, as long as they are accessible to those most likely to have missing or inaccurate records. Any approach to improve a population’s engagement with medical record improvement will depend on public trust in healthcare record systems to protect their data, as highlighted by a recent Swedish survey.^31^

A population-based approach is an effective invitation strategy for a community screening programme located in areas of high socioeconomic deprivation with high smoking rates. Our modelling suggests the optimal targeted invitation approach includes ever-smokers based on the primary care recorded live and historic smoking status and individuals with absent smoking codes. Targeted approaches reduce costs and avoid potential harm to never-smokers, but may miss a proportion of higher risk ever-smokers and individuals with screen-detected lung cancer. This issue could be further mitigated by improving the accuracy of primary care recorded smoking codes.

## Data Availability

Data are available upon reasonable request.
