# Supplementary material for: Invitation strategies and participation in a community-based lung cancer screening programme located in areas of high socioeconomic deprivation
Source: Thorax. 2023 Aug 16;79(1):58–67. doi: 10.1136/thorax-2023-220001 (PMC10803959; doi:10.1136/thorax-2023-220001)
Supplement: Supplementary data [file thorax-2023-220001supp001.pdf]

Supplementary appendix

Table S1. Smoking code clusters, as per NHS Digital business rules

ClusterName	SNOMED concept ID	Term
EXSMOK_COD	1092031000000108	Ex-smoker amount unknown (finding)
EXSMOK_COD	1092041000000104	Ex-very heavy smoker (40+/day) (finding)
EXSMOK_COD	1092071000000105	Ex-heavy smoker (20-39/day) (finding)
EXSMOK_COD	1092091000000109	Ex-moderate smoker (10-19/day) (finding)
EXSMOK_COD	1092111000000104	Ex-light smoker (1-9/day) (finding)
EXSMOK_COD	1092131000000107	Ex-trivial smoker (<1/day) (finding)
EXSMOK_COD	160617001	Stopped smoking (finding)
EXSMOK_COD	160620009	Ex-pipe smoker (finding)
EXSMOK_COD	160621008	Ex-cigar smoker (finding)
EXSMOK_COD	160625004	Date ceased smoking (observable entity)
EXSMOK_COD	228486009	Time since stopped smoking (observable entity)
EXSMOK_COD	266921000	Ex-trivial cigarette smoker (<1/day) (finding)
EXSMOK_COD	266922007	Ex-light cigarette smoker (1-9/day) (finding)
EXSMOK_COD	266923002	Ex-moderate cigarette smoker (10-19/day) (finding)
EXSMOK_COD	266924008	Ex-heavy cigarette smoker (20-39/day) (finding)
EXSMOK_COD	266925009	Ex-very heavy cigarette smoker (40+/day) (finding)
EXSMOK_COD	266928006	Ex-cigarette smoker amount unknown (finding)
EXSMOK_COD	281018007	Ex-cigarette smoker (finding)
EXSMOK_COD	360890004	Intolerant ex-smoker (finding)
EXSMOK_COD	360900008	Aggressive ex-smoker (finding)
EXSMOK_COD	48031000119106	Ex-smoker for more than 1 year (finding)
EXSMOK_COD	492191000000103	Ex roll-up cigarette smoker (finding)
EXSMOK_COD	53896009	Tolerant ex-smoker (finding)
EXSMOK_COD	735112005	Date ceased using moist tobacco (observable entity)
EXSMOK_COD	735128000	Ex-smoker for less than 1 year (finding)
EXSMOK_COD	8517006	Ex-smoker (finding)
LSMOK_COD	134406006	Smoking reduced (finding)
LSMOK_COD	160603005	Light cigarette smoker (1-9 cigs/day) (finding)
LSMOK_COD	160604004	Moderate cigarette smoker (10-19 cigs/day) (finding)
LSMOK_COD	160605003	Heavy cigarette smoker (20-39 cigs/day) (finding)
LSMOK_COD	160606002	Very heavy cigarette smoker (40+ cigs/day) (finding)
LSMOK_COD	160612007	Keeps trying to stop smoking (finding)
LSMOK_COD	160613002	Admitted tobacco consumption possibly untrue (finding)
LSMOK_COD	160616005	Trying to give up smoking (finding)
LSMOK_COD	160619003	Rolls own cigarettes (finding)
LSMOK_COD	203191000000107	Wants to stop smoking (finding)
LSMOK_COD	225934006	Smokes in bed (finding)
LSMOK_COD	230056004	Cigarette consumption (observable entity)
LSMOK_COD	230057008	Cigar consumption (observable entity)
LSMOK_COD	230058003	Pipe tobacco consumption (observable entity)
LSMOK_COD	230059006	Occasional cigarette smoker (finding)
LSMOK_COD	230060001	Light cigarette smoker (finding)
LSMOK_COD	230062009	Moderate cigarette smoker (finding)
LSMOK_COD	230063004	Heavy cigarette smoker (finding)
LSMOK_COD	230064005	Very heavy cigarette smoker (finding)
LSMOK_COD	230065006	Chain smoker (finding)
LSMOK_COD	266918002	Tobacco smoking consumption (observable entity)
LSMOK_COD	266920004	Trivial cigarette smoker (less than one cigarette/day) (finding)
LSMOK_COD	266929003	Smoking started (finding)
LSMOK_COD	308438006	Smoking restarted (finding)
LSMOK_COD	394871007	Thinking about stopping smoking (finding)
LSMOK_COD	394872000	Ready to stop smoking (finding)
LSMOK_COD	394873005	Not interested in stopping smoking (finding)
LSMOK_COD	401159003	Reason for restarting smoking (observable entity)
LSMOK_COD	413173009	Minutes from waking to first tobacco consumption (observable entity)
LSMOK_COD	428041000124106	Occasional tobacco smoker (finding)
LSMOK_COD	446172000	Failed attempt to stop smoking (finding)
LSMOK_COD	449868002	Smokes tobacco daily (finding)
LSMOK_COD	56578002	Moderate smoker (20 or less per day) (finding)
LSMOK_COD	56771006	Heavy smoker (over 20 per day) (finding)
LSMOK_COD	59978006	Cigar smoker (finding)
LSMOK_COD	65568007	Cigarette smoker (finding)
LSMOK_COD	77176002	Smoker (finding)
LSMOK_COD	82302008	Pipe smoker (finding)
LSMOK_COD	836001000000109	Waterpipe tobacco consumption (observable entity)
NSMOK_COD	221000119102	Never smoked any substance (finding)
NSMOK_COD	266919005	Never smoked tobacco (finding)

Supplement S2.

Comparison of live smoking codes from primary care and self-reported smoking status is shown in Table S2a, and live & historical codes in Table S2b. If both live and historical codes were used to define primary care smoking status, the proportion classified as ever-smokers increased from 52% to 63% (n=21,451/34,261). The overall rate of discordance between primary care recorded smoking status and self-reported smoking status in those contacting the LHC service was 12% (n=1,861/15,107). Discordance was 11% (n=1,123/10,491) and 16% (n=738/4,616) in primary care recorded ever- and never-smokers respectively. Discordance was highest in those classified as an ever-smoker based on historical codes only (their 'live' status was never-smoker), with 50% (n=896/1,805) self-reporting being a never-smoker, compared to only 2.6% (n=227/8,686) in those with a live ever-smoking status.

We also compared the presence of any ever-smoking status codes (live or historical) in primary care with self-reported ever-smoking status and discordance varied from 6.2% to 30% (median 11%). The proportion of respondents historically coded as never-smokers who self-reported being ever-smokers ranged from 1.3% to 11% between practices (median 4.5%).

Table S2a. Comparison of live primary care smoking statuses (L) with self-reported smoking status among invitees with an available primary care record who responded to invitation.

	**Overall** N = 15,545	**GP code: ever-smoker (L)** N = 8,686	**GP code: never-smoker (L)** N = 6,421	**Absent code** N = 438
**Self-reported ever-smoker**, n (%)	10,315 (66%)	8,459 (97%)	1,647 (26%)	209 (48%)
**Self-reported never-smoker**, n (%)	5,230 (34%)	227 (2.6%)	4,774 (74%)	229 (52%)

Table S2b. Comparison of live and historical primary care smoking statuses (L&H) with self-reported smoking status among invitees with an available primary care record who responded to invitation.

	**Overall** N = 15,545	**Ever-smoker (L&H GP code)** N = 10,491	**Never-smoker (L&H GP code)** N = 4,616	**Absent code** N = 438
**Self-reported ever-smoker**, n (%)	10,315 (66%)	9,368 (89%)	738 (16%)	209 (48%)
**Self-reported never-smoker**, n (%)	5,230 (34%)	1,123 (11%)	3,878 (84%)	229 (52%)

Table S3. Comparison of the impact of three invitation strategies along the screening pathway, and key screening outcomes, compared to population invitation strategy used in North & East Manchester, among invitees with an available primary care record.
(L) refers to live smoking status codes; (H) refers to historical smoking status codes.

Invitation Strategy	Population	Ever-smoker (L&H) & Absent	Ever-smoker (L&H)	Ever-smoker (L)
LHC invitations sent	34,261	23,259	22,451	17,895
Eligible respondents (self-reported ever-smokers)	10,315	9,577	9,368	8,459
LHC bookings	9,322	8,739	8,574	7,747
LHC attendances	8,590	8,101	7,955	7,187
Screen-eligible	4,396	4,364	4,225	4,160
Participants screened	4,330	4,298	4,299	4,096
Lung cancers detected	140	138	136	136
Invitations per lung cancer detected	244.7	157.7	142.8	131.6
LHCs per lung cancer detected	61.4	58.5	53.1	52.8
LDCT screenees per lung cancer detected	30.9	31.1	30.1	30.1
